# Clinical outcomes of FOLFIRINOX in locally advanced pancreatic cancer: A single center experience

**DOI:** 10.1097/MD.0000000000013592

**Published:** 2018-12-14

**Authors:** Jongchan Lee, Jong-chan Lee, Mark A. Gromski, Hyoung Woo Kim, Jinwon Kim, Jaihwan Kim, Jin-Hyeok Hwang

**Affiliations:** a Department of Internal Medicine, Seoul National University, College of Medicine, Seoul National University Bundang Hospital, Seongnam, Korea; b Department of Internal Medicine, Division of Gastroenterology, Indiana University School of Medicine, Indianapolis, Indiana, USA; c Department of Internal Medicine, Chungbuk National University, College of Medicine, Chungbuk National University Hospital, Cheongju, Korea.

**Keywords:** dose intensity, FOLFIRINOX, locally advanced pancreatic cancer, R0 resection, toxicity

## Abstract

Systemic chemotherapy or chemoradiotherapy is the initial primary option for patients with locally advanced pancreatic cancer (LAPC). This study analyzed the effect of FOLFIRINOX and assessed the factors influencing conversion to surgical resectability for LAPC.

Sixty-four patients with LAPC who received FOLFIRINOX as initial chemotherapy were enrolled retrospectively. Demographic characteristics, tumor status, interval/dosage/cumulative relative dose intensity (cRDI) of FOLFIRINOX, conversion to resection, and clinical outcomes were reviewed and factors associated with conversion to resectability after FOLFIRINOX were analyzed.

After administration of FOLFIRINOX (median 9 cycles, 70% of cRDI), the median patient overall survival (OS) was 17.0 months. Fifteen of 64 patients underwent surgery and R0 resection was achieved in 11 patients. During a median follow-up time of 9.4 months after resection, cumulative recurrence rate was 28.5% at 18 months after resection. The estimated median OS was significantly longer for the resected group (>40 months vs 13 months). There were no statistical differences between the resected and non-resected groups in terms of baseline characteristics, tumor status and hematologic adverse effects. The patients who received standard dose of FOLFIRINOX had higher probability of subsequent resection compared with patients who received reduced dose, although cRDIs did not differ between groups.

FOLFIRINOX is an active regimen in patients with LAPC, given acceptable resection rates and promising R0 resection rates. Additionally, our data demonstrate it is advantageous for obtaining resectability to administer FOLFIRINOX without dose reduction.

## 1 Introduction

Pancreatic ductal adenocarcinoma (PDA) is associated with a dismal prognosis, with a 5-year survival rate of 6%.^[1]^ This disease is one of the leading causes of cancer-related mortality worldwide, and surgical resection is the only modality that can lead to long-term survival.^[2]^ However, approximately 80% of patients are not surgical candidates, due to distant metastases or vascular invasion at the time of diagnosis.^[2]^ About 1/3^rd^ of patients with pancreatic cancers display unresectable localized disease, and the median overall survival (OS) is approximately 9 to 12 months for this cohort.^[3,4]^

In the past, most patients with locally advanced pancreatic cancer (LAPC) could not undergo resection, even after chemotherapy and chemoradiotherapy, due to lack of effective chemotherapeutic regimens and local control modalities. As a result, LAPC was primarily treated with palliation without curative intent. Since it was discovered that FOLFIRINOX (fluorouracil, leucovorin, irinotecan, and oxaliplatin) was an active regimen that improved survival in patients with metastatic pancreatic cancer up to 11.1 months, there have been several attempts to use FOLFIRINOX as neoadjuvant chemotherapy for LAPC.^[5–9]^ A recent meta-analysis of 13 studies found that FOLFIRINOX could give a quarter of patients with LAPC a chance to undergo subsequent resection and provide a substantially longer median survival time of 24.2 months.^[10]^ However, it remains unclear which factors determine resectability after FOLFIRINOX administration in patients with LAPC.

In this study, we report our institutional investigation of patients with LAPC who were treated with FOLFIRINOX and assess factors that influence conversion to resectability with neoadjuvant FOLFIRINOX in LAPC.

## 2 Materials and methods

### 2.1 Patient and data sources

This is a retrospective study of patients with LAPC who were treated with neoadjuvant FOLFIRINOX at Seoul National University Bundang Hospital between April 2012 and June 2016. This study was undertaken with the approval of the Institutional Review Board. We reviewed patient electronic medical records and subsequently followed their respective clinical courses.

Patients with LAPC who received FOLFIRINOX as 1st-line treatment were included. They were all diagnosed with PDA by histology or cytology. The decision of resectability was made through careful multi-disciplinary pancreatic cancer board review using pancreatic protocol CT, endoscopic ultrasound, magnetic resonance imaging (MRI), and PET-CT based on the National Comprehensive Cancer Network guidelines.

FOLFIRINOX doses were planned according to the protocol used in the ACCORD11/PRODIGE4 trial.^[5]^ FOLFIRINOX was administered in 14-day cycles. Oxaliplatin was given at a dose of 85 mg/m^2^ over 2 hours, leucovorin at 400 mg/m^2^ over 2 hours, irinotecan at 180 mg/m^2^ over 90 minutes, and 5-FU as a bolus of 400 mg/m^2^. After that, 5-FU was continuously administered with 2400 mg/m^2^ over 46 hours. Use of granulocyte-colony stimulating factor (G-CSF) and dose adjustments were decided at the discretion of the treating physician, based on development and severity of neutropenia.

FOLFIRINOX administration proceeded until disease progression was recognized, surgical resection was achieved, or unacceptable adverse effects were encountered. The evaluation of response to FOLFIRINOX was conducted every 8 to 12 weeks by contrast-enhanced CT (CECT). When additional assessment was needed, MRI or PET-CT was used. Whenever response is evaluated using CECT, resectability was re-evaluated through a multi-disciplinary pancreatic cancer board review. The patients who did not obtain resectability, despite adequate administration of FOLFIRINOX, received stereotactic body radiotherapy (SBRT). The SBRT was delivered in 5 fractions to a total dose of 33 Gy over 10 days. The target goal for the planning target volume (PTV) was that ≥ 90% of PTV receives 100% of the prescription dose (33 Gy) and no more than 1 mL inside PTV exceeds 130% of the prescribed dose. All patients received volumetric modulated arc therapy on the TrueBeam system (Varian Medical Systems, Inc, Palo Alto, CA).

We analyzed probable related factors to determine subsequent resectability, such as sex, age, eastern cooperative oncology group performance status (ECOG PS), body mass index (BMI), abdominal muscle area (cm^2^), initial CA19-9 level, location and size of tumors, and involved vessels. The abdominal muscle area, composed of paraspinal and abdominal wall muscles at the 3rd lumbar level, was measured using the SliceOmatic program (Tomovision, Canada).

We also analyzed chemotherapy cycles, duration, dosage reduction, cumulative relative dose intensity (cRDI) of FOLFIRINOX, and tumor response. The cRDI is the percentage of the administered cumulative dose to the standard cumulative dose in a certain period. If FOLFIRINOX is administered regularly on time for 2 weeks without dose reduction, cRDI is 100%. We calculated cRDI using a java program that was developed by our group (http://www.hwang-lab.com/02_rdicalc).^[11]^ The response evaluation criteria in Solid Tumors 1.1 (RECIST 1.1) guideline was used for evaluation of the response to the chemotherapy.

Evaluation of FOLFIRINOX toxicity was performed using the National Cancer Institute Common Toxicity Criteria. R0 resection was defined as tumor margins being free of tumor. The OS was designated as date of diagnosis to the date of death.

### 2.2 Statistical methods

Baseline and demographic characteristics were summarized as median and range for continuous variables and as frequencies for categorical variables. Comparisons were performed with the Mann–Whitney *U* [for continuous variables] and Fisher exact test or Chi-square test [for categorical variables]. A multivariate logistic regression model was performed to assess predictors associated with resectability. We consider *P* value < .05 to indicate statistical significance. The OS was calculated using the Kaplan–Meier method with corresponding 2-sided 95% confidence intervals (CIs). Statistical analyses were performed using STATA version 14.0 (Stata Corp, College Station, TX).

## 3 Results

### 3.1 Baseline patients’ characteristics

Between April 2012 and June 2016, a total of 64 patients with LAPC were treated with FOLFIRINOX at our hospital. Clinical follow-up was accrued through June 2017.

Baseline characteristics are summarized in Table 1. Among 64 patients, 15 patients (23.4%) underwent resection after FOLFIRINOX therapy (resected group) and the remaining 49 patients were categorized in the non-resected group.

**Table 1:**
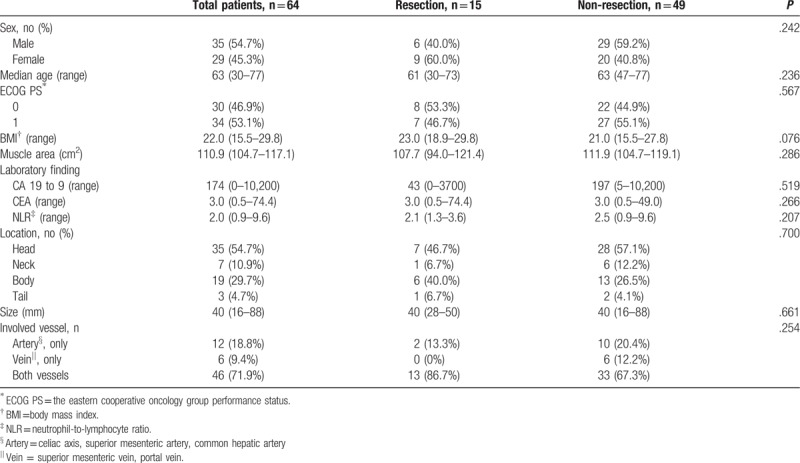
Patients’ baseline characteristics, n = 64.

The median patient age was 63 years (30–77 years), and 54.7% of patients were male. All patients had an ECOG PS of 0 or 1, and 19 patients (29.7%) required biliary decompression with bypass procedures such as endoscopic retrograde cholangiography and percutaneous transhepatic biliary drainage to relieve biliary obstruction at the start of FOLFIRINOX. Pancreatic cancer was located at head and neck in 42 patients (65.6%), and the median tumor size was 40 mm (range, 16–88 mm). There were no significant differences with regard to sex, age, ECOG PS, BMI, abdominal muscle area, tumor markers, tumor location, tumor size, and major vessel involvement between resected and non-resected groups (*P* > .05) (Table 1).

### 3.2 FOLFIRINOX treatment and outcomes

**Figure 1:**
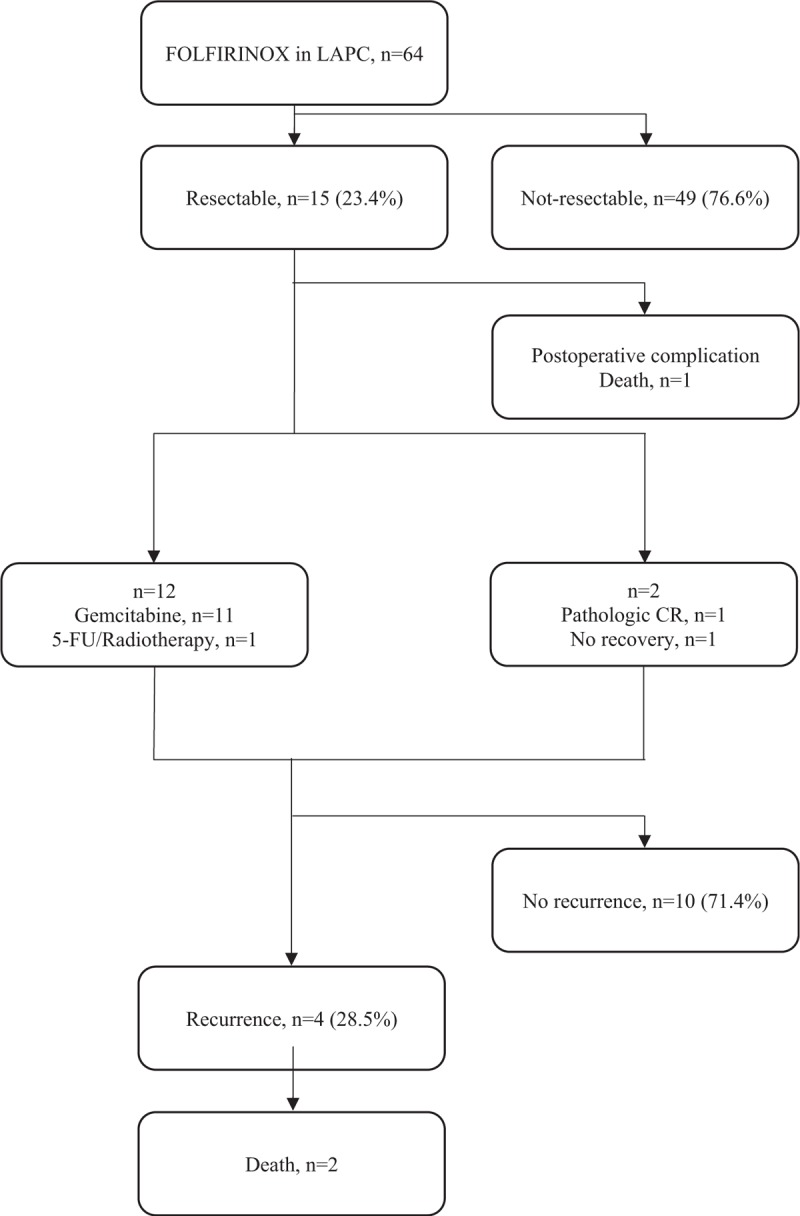
Flowchart showing outcomes of patients treated with FOLFIRINOX for locally advanced pancreatic cancer. Of the 64 patients treated with FOLFIRINOX for locally advanced pancreatic cancer, 15 patients had acquired resectability and underwent surgery. One patient, who had systemic erythematous lupus, died of acute mesenteric artery thrombosis after surgery. Among 14 patients except 1 who died, 12 patients received adjuvant chemotherapy (gemcitabine-based chemotherapy in 11 patients) and 2 patients did not receive adjuvant chemotherapy. One of the 2 patients who did not receive adjuvant chemotherapy was found to have pathologic complete remission and the other had not yet recovered after surgery. Pancreatic cancer recurred in 4 patients of 14 patients and 2 patients died.

Figure 1 provides a flowchart of patient response to therapy. A total of 556 cycles of FOLFIRINOX were administered to 64 patients (median 9 cycles [1–20]), and the median duration of FOLFIRINOX was 6.0 months (0.4–12.2 months). The number of cycles or duration of FOLFIRINOX was not significantly different between the groups (*P* > .05, Table 2).

**Table 2:**
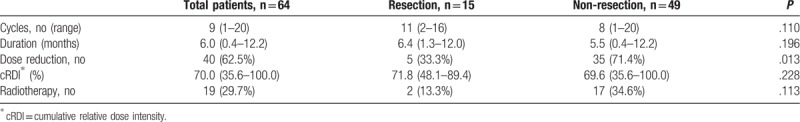
Treatment: administration of FOLFIRINOX and chemoradiation.

Forty patients (62.5%) required dose reductions. Fewer patients required dose reduction in the resected group than in the non-resected group (33.3% (5/15) vs 71.4% (35/49), respectively, *P* = .013), although cRDIs did not differ between groups (71.8% vs 69.6%, respectively). Nineteen patients were not suited for resection and required further SBRT, and resection was achieved in 2 additional patients after SBRT (Table 2). The overall response and disease control rates were much higher in the resected group than in the non-resected group (32.7% and 8.2% vs. 100% and 66.7%, *P* < .001, respectively) (Table 3).

**Table 3:**
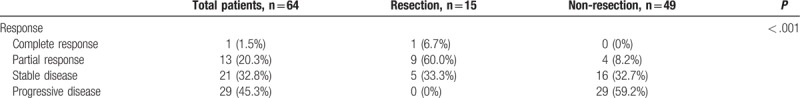
Efficacy results after FOLFIRINOX.

Adverse effects are summarized in Table 4. There were no statistical differences between the resected and non-resected groups in terms of adverse effects, although the non-resected group showed a higher rate of non-hematologic adverse effects. Ten out of 64 patients (15.6%) experienced febrile neutropenia, which did not differ between the groups. However, grade 4 febrile neutropenia was observed in 3 patients of the non-resected group, among whom 2 experienced mortality due to sepsis, even after schedule and dose modification of FOLFIRINOX and use of G-CSF.

**Table 4:**
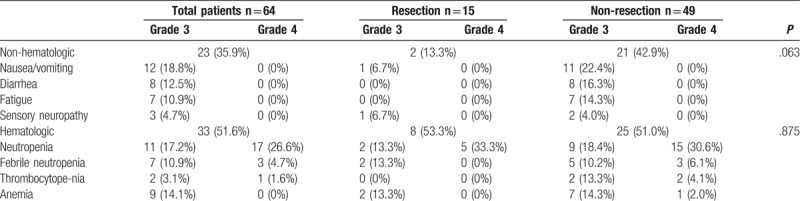
Grade 3 to 4 toxicities related to FOLFIRINOX treatment [n (%)].

### 3.3 Surgical and long-term outcomes

Table 5 summarizes the clinical data for 15 patients who underwent surgery for LAPC after neoadjuvant FOLFIRINOX. The median time to resection was 6.7 months (3.2–14.3 months). Among the 15 patients, 8 patients received pancreaticoduodenectomy and 7 received distal pancreatectomy. Eleven patients achieved R0 resection following FOLFIRINOX therapy (73.3%), 9 patients had lymph nodes metastases, and 1 patient achieved pathologic complete response. Twelve patients received adjuvant chemotherapy (gemcitabine-based chemotherapy in 11 patients). One patient, who displayed a systemic erythematous lupus disorder, died of postoperative complication of acute mesenteric artery thrombosis, despite heparinization after surgery.

**Table 5:**
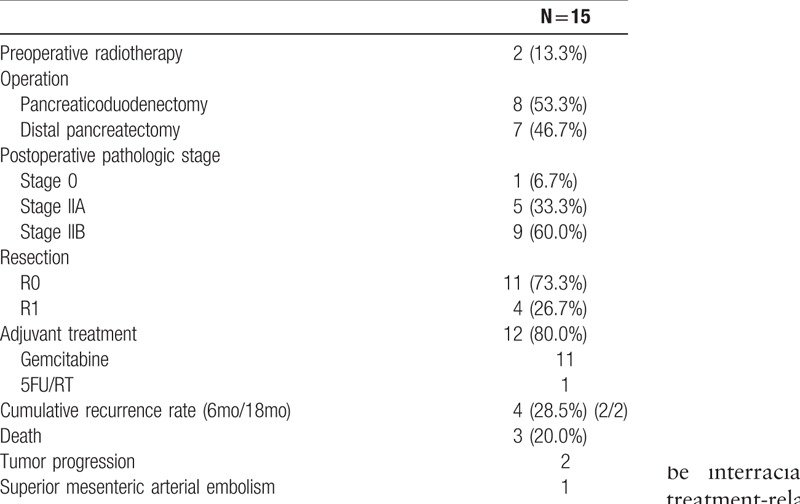
Summary of surgical and pathologic outcomes.

During a median follow-up time of 9.4 months after resection (1.8–35.5 months) and 23.1 months after diagnosis (15.0–46.1 months), 4 patients observed recurrences in 3.3, 5.1, 13.9, and 15.7 months, respectively, after resection. The cumulative recurrence rate was 14.3% at 6 months and 28.5% at 18 months after resection. Among this cohort, 2 patients remained alive 20.2 months and 35.5 months, respectively, after resection (26.3 and 43.4 months after diagnosis, respectively). Ten patients did not experience recurrence during the median follow-up time of 20.3 months after resection (10.7 – 35.5 months) and 23.9 months after diagnosis (15.0–46.1 months).

**Figure 2:**
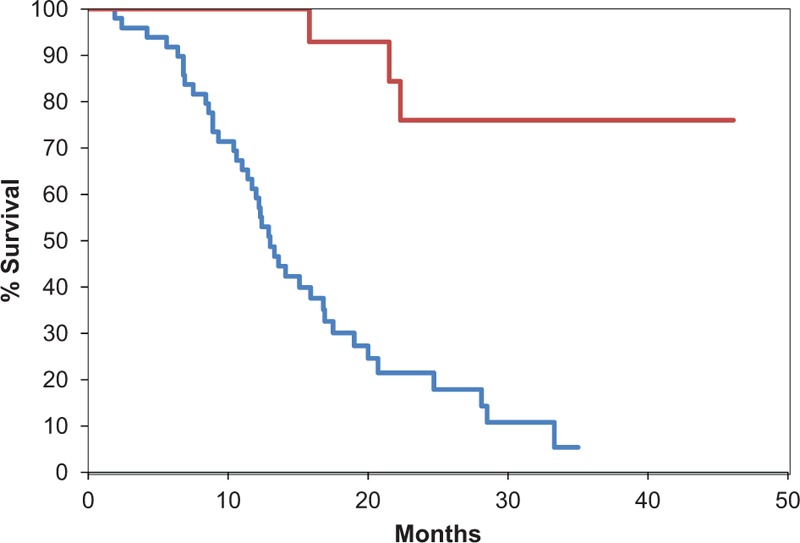
Kaplan–Meier survival estimates of overall survival of groups with resected (*red line*) and unresected (*blue line*) pancreatic cancer after FOLFIRINOX treatment. The median overall survival (OS) for all enrolled patients was 17.0 months (1.9–46.1 months). The median OS of the resected group was unable to be accurately calculated because > 50% of patients remain alive (> 40 months) but was clearly better than that of the non-resected group (13.0 months). OS = overall survival.

The median OS for all enrolled patients was 17.0 months (1.9–46.1 months). The 1-year survival rate for all patients was 70.3% and the 2-year survival rate was 34.7%. The median OS of the resected group was unable to be accurately calculated because > 50% of patients remain alive (> 40 months) but was clearly better than that of the non-resected group (13.0 months) (Fig. 2).

### 3.4 Predictors associated with resectability

As shown in Table 6, the presence of dose reduction was associated with the failure to convert to resectability in univariate and multivariate logistic regression. The patients who received reduced dose intensity in each cycle had lower probability of subsequent resection compared with patients who received standard dose of FOLFIRINOX (*P* = .024), although cRDIs did not display statistically significant differences between the groups (*P* = .639). None of the other factors included in the regression analyses were associated with resectability.

**Table 6:**
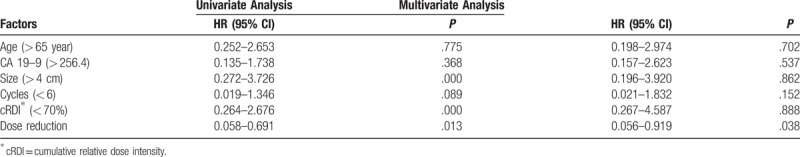
Predictable factors for resectability after FOLFIRINOX.

## 4 Discussion

This study presents outcomes of a large series of patients with LAPC who received neoadjuvant FOLFIRINOX chemotherapy as a naïve treatment in a single institution. Our data show that 23.4% of patients with LAPC became resectable and surgical candidates, and R0 resection was achieved in 73.3% of patients after adequate FOLFIRINOX chemotherapy, in agreement with a report from the western countries (25.9% and 78.4%, respectively).^[10]^ Furthermore, our data demonstrate that, for obtaining resectability, it is advantageous to administer FOLFIRINOX without dose reduction, if tolerated by the patient.

According to the meta-analysis released in 2012, after gemcitabine-based neoadjuvant chemotherapy in patients with LAPC, resection was performed in 27% of cases and the R0 resection ratio was only 23%.^[12]^ In contrast, our data show that around 1/4th of patients with LAPC can undergo resection, of which about 75% are R0 resections, and this is in accordance with a recent systematic review of FOLFIRINOX.^[10]^ In agreement with our results, patients with LAPC who underwent surgery after neoadjuvant FOLFIRINOX can expect median OS of > 3 years, which is much longer than the median OS (∼24 months) of patients with resected pancreatic cancer.^[10]^ Therefore, the treatment strategy for patients with LAPC should be changed to curative intent, rather than palliation, in the era of FOLFIRINOX.

While methodologies and influential factors towards achieving higher resection rate have not been well described, we found that patients who maintain planned dose of FOLFIRINOX are more likely to convert to displaying a resectable tumor than those who do not, despite similar total administered dosage of FOLFIRINOX during treatments. In other words, interval modification may confer more resectability outcomes than dose modification, and we suggest to 1st consider interval modification, and whether dose or schedule should be inevitably modified due to variable reasons. Our results are supported by a recent study, which found that increasing the number of full-dose neoadjuvant FOLFIRINOX treatments was significantly associated with increased survival.^[13]^

FOLFIRINOX displays adverse effects. Recently published eastern data show much higher rates of grade 3 or 4 febrile neutropenia in comparison with a report from the Western countires (15.6% of our institution / 22.2% of Japanese patients vs. 5% of Western patients).^[10,14,15]^ In this study, 2 patients died of neutropenic fever and accompanying complications after FOLFIRINOX, despite the use of G-CSF and adequate dose and interval modification. This observation suggests that there may be interracial differences, especially since there were no treatment-related mortalities (0/355) in a recent systematic review from Western country.^[10]^ Clinicians face a dilemma of how to maintain dose intensity while avoiding febrile neutropenia in patients with LAPC who are treated with FOLFIRINOX, especially in Asian populations. We do not observe febrile neutropenia after the use of pegylated G-CSF. Based on our experience and recent study showing preventive effect of pegylated G-CSF against neutropenia during FOLFIRINOX,^[16]^ we would suggest that pegylated G-CSF can be an option for keeping FOLFIRINOX dose intensity, although it requires further investigation.

The role of radiotherapy in patients with LAPC remains a controversial issue. Nineteen patients had SBRT with FOLFIRINOX and 2 patients achieved resectability (2/19, 10%). This suggests that SBRT increases resectability when resectable status is not achieved, even after adequate administration of FOLFIRINOX in patients with LAPC. Although SBRT has theoretical advantages, such as short duration of radiation, delivery of more ablative doses to the tumor, and minimization of interrupting systemic chemotherapy, the role of SBRT in patients with LAPC requires further clarification in the future.^[17]^

This study was limited to a single center retrospective analysis; however, our data can be considered valuable because 64 patients with LAPC who received FOLFIRINOX from a single center represent one of the largest cohorts in the world, particularly in Asian populations. Although we discovered that FOLFIRINOX without dose modification provides a higher rate of subsequent resectability in patients with LAPC, prospective studies should be conducted to further clarify this finding.

In conclusion, FOLFIRINOX should be considered as an active regimen in patients with LAPC, since this therapy provides an acceptable resection rate and promising R0 resection rate. If patients tolerate this treatment, the administration of FOLFIRINOX without dose reduction is associated with achieving resectability.

## Acknowledgments

These data were presented in part at the ASCO Gastrointestinal Cancer Symposium 2017, San Francisco, California.

## Author contributions

**Conceptualization:** Jaihwan Kim, Jin-Hyeok Hwang.

**Data curation:** Jongchan Lee, Jong-chan Lee, Hyoung Woo Kim.

**Formal analysis:** Jongchan Lee, Jin-Hyeok Hwang.

**Investigation:** Jongchan Lee, Hyoung Woo Kim, Jin-Hyeok Hwang.

**Methodology:** Jongchan Lee, Jaihwan Kim, Jin-Hyeok Hwang.

**Project administration:** Jongchan Lee.

**Resources:** Jinwon Kim, Jaihwan Kim.

**Software:** Jong-chan Lee.

**Supervision:** Jin-Hyeok Hwang.

**Validation:** Jong-chan Lee, Jin-Hyeok Hwang.

**Visualization:** Jongchan Lee.

**Writing – original draft:** Jongchan Lee.

**Writing – review & editing:** Jongchan Lee, Mark A. Gromski, Jin-Hyeok Hwang.

## References

[1] Siegel R Naishadham D Jemal A Cancer statistics, 2012. CA Cancer J Clin 2012;62:10–29. 22237781 10.3322/caac.20138

[2] Geer RJ Brennan MF Prognostic indicators for survival after resection of pancreatic adenocarcinoma. Am J Surg 1993;165:68–72. discussion 72-63. 8380315 10.1016/s0002-9610(05)80406-4

[3] Stathis A Moore MJ Advanced pancreatic carcinoma: current treatment and future challenges. Nat Rev Clin Oncol 2010;7:163–72. 20101258 10.1038/nrclinonc.2009.236

[4] Jason EF Lawrence SB Shaunagh M FOLFIRINOX in locally advanced pancreatic cancer: the Massachusetts general hospital cancer center experience. Oncologist 2013;18:543–8. 23657686 10.1634/theoncologist.2012-0435 PMC3662845

[5] Conroy T Desseigne F Ychou M FOLFIRINOX versus gemcitabine for metastatic pancreatic cancer. N Engl J Med 2011;364:1817–25. 21561347 10.1056/NEJMoa1011923

[6] Cristina R Ferrone Giovanni Marchegiani Theodore S Hong Radiological and surgical implications of neoadjuvant treatment with FOLFIRINOX for locally advanced and borderline resectable pancreatic cancer. Ann Surg 2015;261:12–7. 25599322 10.1097/SLA.0000000000000867 PMC4349683

[7] Alysadra L Kathleen C Doublas BE Management of borderline resectable pancreatic cancer. Surg Oncol Clin N Am 2010;19:359–70. 20159519 10.1016/j.soc.2009.11.006

[8] Petrelli F Coinu A Borgonovo K FOLFIRINOX-based neoadjuvant therapy in borderline resectable or unresectable pancreatic cancer: a meta-analytical review of published studies. Pancreas 2015;44:515–21. 25872127 10.1097/MPA.0000000000000314

[9] Hosein PJ Macintyre J Kawamura C A retrospective study of neoadjuvant FOLFIRINOX in unresectable or borderline-resectable locally advanced pancreatic adenocarcinoma. BMC Cancer 2012;12:199. 22642850 10.1186/1471-2407-12-199 PMC3404979

[10] Mustafa S Berend RB Eran S FOLFIRINOX for locally advanced pancreatic cancer: a systematic review and patient-level meta-analysis. Lancet Oncol 2016;17:801–10. 27160474 10.1016/S1470-2045(16)00172-8 PMC5527756

[11] Hwang JH Kim JH Lee JC RDI Calculator for combination chemotherapy. Hwang-lab.com 2015. http://www.rdicalc.com (Accessed 30 June 2016).

[12] Andriulli A Festa V Botteri E Neoadjuvant/preoperative gemcitabine for patients with localized pancreatic cancer: a meta-analysis of prospective studies. Ann Surg Oncol 2012;19:1644–62. 22012027 10.1245/s10434-011-2110-8

[13] Moh’d Khushman Naomi Dempsey Jennifer Cudris Maldonado Full dose neoadjuvant FOLFIRINOX is associated with prolonged survival in patients with locally advanced pancreatic adenocarcinoma. Pancreatology 2015;15:667–73. 26412296 10.1016/j.pan.2015.08.010

[14] Jong-chan Lee Jin Won Kim Soyeon Ahn Optimal dose reduction of FOLFIRINOX for preserving tumour response in advanced pancreatic cancer: using cumulative relative dose intensity. Eur J Cancer 2017;76:125–33. 28324747 10.1016/j.ejca.2017.02.010

[15] Takuji Okusaka Masafumi Ikeda Akira Fukutomi Phase II study of FOLFIRINOX for chemotherapy-naïve Japanese patients with metastatic pancreatic cancer. Cancer Sci 2014;105:1321–6. 25117729 10.1111/cas.12501 PMC4462360

[16] de W Marsh R Mark ST Primary systemic therapy in resectable pancreatic ductal adenocarcinoma using mFOLFIRINOX: A pilot study. J Surg Oncol 2017;1–9. 10.1002/jso.24872 PMC5890423 29044544

[17] Thomas B Brunner, Ursula Nestle, Anca-Ligia Grosu et al. SBRT in pancreatic cancer: what is the therapeutic window? Radiother Oncol 2015;114:109–16. 25466369 10.1016/j.radonc.2014.10.015

